# Changes in Australian community perceptions of non-communicable disease prevention: a greater role for government?

**DOI:** 10.1186/s12889-021-12159-9

**Published:** 2021-11-15

**Authors:** Anne C. Grunseit, Eloise Howse, Erika Bohn-Goldbaum, Jo Mitchell, Adrian E. Bauman

**Affiliations:** 1 The Australian Prevention Partnership Centre, Prevention Research Collaboration, Sydney School of Public Health, Level 6, Charles Perkins Centre, University of Sydney, Camperdown, NSW 2006 Australia; 2 The Australian Prevention Partnership Centre, The Sax Institute, Level 3, 30C Wentworth St, Glebe, NSW 2037 Australia

**Keywords:** Australia, Public opinion, Attitudes, Prevention, Non-communicable diseases, Policy

## Abstract

**Background:**

Monitoring trends in community opinion can identify critical opportunities to implement upstream health policies or interventions. Our study examines change and demographic modifiers of change in community perceptions of government intervention for prevention of lifestyle-related chronic disease across two time points in Australia.

**Methods:**

Data were drawn from the 2016 (*n* = 2052) and 2018 (*n* = 2601) waves of a nationally representative cross-sectional telephone survey, ‘AUSPOPS’. Survey questions gauged perceptions of government intervention for health in general, peoples’/organizations’ role in maintaining health (e.g., parents, government) and support for specific health interventions (e.g., taxing soft drink).

Bivariate and multivariate regression models tested for change between the two surveys, adjusted for demographic characteristics. Models with interactions between survey wave and demographic variables tested for differential change. One-tailed variance ratio tests examined whether opinions had become more polarized in 2018 compared with 2016.

**Results:**

The large, significant increase observed in the perceived size of the role that government has in maintaining people’s health was uniform across demographic subpopulations. The role for employers and private health insurers was also perceived to be larger in 2018 compared with 2016, but the degree of change varied by gender, age and/or socioeconomic status. Support for some government interventions (e.g., taxing soft drinks) increased among specific demographic subgroups whilst exhibiting no overall change. Opinion was more polarized on general attitudes to government intervention for population health in 2018 compared to 2016, despite little change in central tendency.

**Conclusions:**

Opportunities may exist to implement government health-promoting policies (e.g., taxing soft drinks), although advocacy may be needed to address the concerns of less supportive subpopulations. Attitudes on government intervention in general may be becoming more polarized; future research examining the association of such changes with exposure to different information sources could inform communication strategies for future health policy change.

**Supplementary Information:**

The online version contains supplementary material available at 10.1186/s12889-021-12159-9.

## Background

Despite the considerable effort to address the burden of non-communicable chronic diseases (NCDs) such as diabetes and cardiovascular disease, the prevalence of risk factors for NCDs, with the possible exception of tobacco use, have changed little over the past few decades and remain high [1]. Overweight and obesity and their health consequences pose a particular challenge contributing 8.4% to the burden of disease in Australia [1]. Although the majority of strategies designed to reduce the lifestyle-related risks leading to NCDs, including overweight/obesity, have been directed towards the individual behavior change, many health promotion advocates [2, 3] and lead health agencies [4, 5] have emphasized the important role of policy and environmental-level change for greater impact. Such actions include introducing fiscal interventions, such as taxes on unhealthy products and/or providing subsidies for healthier options; restricting advertising and promotions; reducing the availability of certain products; changing built environments to support physical activity; and mandatory reformulations of the food supply. Marteau et al. (2019) argue that these more upstream approaches, targeting the physical, economic, social and commercial systems driving behaviors, demonstrate the biggest health gains and should be prioritized by government [6]. A recent Australian consensus statement from leading experts in chronic disease prevention has recommended 11 policy actions which should be prioritized based on previous evidence [7]. For example, the report recommends actions such as a volumetric tax on alcohol as price increases through taxation for alcohol have been shown to have the largest effect in reducing alcohol-related morbidity and mortality. Greater investment in active travel infrastructure and a national physical activity plan is also prioritized as changes in urban design which support modal shift from motorized transport to public and active transport have been demonstrated to significantly increase walking [7]. Other groups such as the World Health Organization have recommended similar interventions, which they argue are cost-effective ‘Best Buys’ for chronic disease prevention [8]. However, calls to make these upstream changes through regulation and structural change can be met with community suspicion about the effectiveness and true purpose of such interventions [9, 10] and reluctance on the part of policymakers due to concerns of public acceptability, amongst other considerations [11, 12]. Yet the evidence base in public health strongly favors descriptive correlational studies, rather than studies examining intervention effectiveness, sustainability and public support – evidence which could assist policymakers to build a case for population-level interventions [13].

According to previous research, the likelihood a policy or intervention will be implemented is the result of a range of intersecting contextual and structural factors [11, 14, 15]. For example, Kingdon (2003) [16] argues that policy action requires the alignment of three “streams”: a clear definition of the (health) problem; a proposal for policy change; and a favorable political context. Health promotion advocates and researchers need to be sensitive to these “policy windows” to capitalize on favorable conditions and implement policy change. Being able to detect such moments is challenging; combining close relationships between researchers and policymakers [15, 17] with good evidence for the likely acceptability to the general public of proposed interventions may address political sensitivity to popular sentiment [9, 18].

Monitoring trends in community opinion can help researchers, advocates and policymakers identify “policy windows” or critical opportunities to build their case for policy change. Analyzing these trends may also shed light on the social and political contextual factors which may influence public opinion. However, few studies have examined changes in opinion towards government intervention for prevention of lifestyle-related disease over time. The majority which do so are based on tobacco control surveys in North America and have identified trends such as increased support for government policies and interventions regulating access to, or sales of, tobacco [19–23]. Similar trends have been found for public support for plain packaging of tobacco products in cohort studies in Australia [24] and the UK [25], removing point-of-sale tobacco advertising and displays in Canada [26] and smoke-free bars among American young people [27], demonstrating reasonable evidence for a causal effect of implementation on support for policy change.

In contrast to what appears to be reasonably uniform trends for tobacco control policy, published research on support for alcohol regulations is mixed. In Canada from 1996 to 2011 [28] and Ireland from 2002 to 2010, [29] support has been shown to depend on the policy mechanism and the degree to which policies on price and availability were already in place. Evidence from Australia on attitudes towards alcohol control policies between 1995 and 2010 showed increasing support beginning in 2004 for policies which restrict availability and accessibility of alcohol [30]. However another study encompassing a later period (2001–2016) [31] showed a drop of 10% in support between 2013 and 2016 following the introduction of restrictions for late night service of alcohol in the capital city of one state (Sydney, New South Wales). Other research suggests that advocacy campaigns may be important interventions for increasing public acceptability of policy changes to reduce risk factors such as sugary drink consumption [32].

Together, these studies have shown that there is complexity in how the community perceives whether government intervention for prevention is appropriate, with community perceptions influenced by degree of implementation in addition to strategy type, target behavior and demographic group, as noted with cross-sectional studies [10, 33, 34]. However, to our knowledge, no published study has reported on changes over time in perceptions about the role of government in promoting health and preventing NCDs more broadly or whether those changes differ by demographic subpopulation. This study will draw on the data collected in the national AUStralian Perceptions Of Prevention Survey (“AUSPOPS”) over two time periods (2016 and 2018) to examine change and modifiers of change in general attitudes to government intervention in addition to specific interventions.

## Methods

### Survey design

The AUSPOPS study is a national repeat cross-sectional general population survey, first undertaken in June–July 2016, to understand how Australian communities perceive government interventions aimed at reducing lifestyle-related chronic disease [10]. The questionnaire was administered a second time in October–November 2018 with a small number of additional questions. Only questions asked in both the 2016 and 2018 surveys are reported here. Ethics approval was obtained from the University of Sydney Human Research Ethics Committee, approval #2016/141.

### Sampling

For both 2016 and 2018 surveys, data collection was contracted to a market research company (Social Research Centre: https://www.srcentre.com.au/) with respondents recruited through a commercial sample provider via Random Digit Dialing covering both landline and mobile phone populations [35]. A stratified (state by region, capital city/non-capital city) landline sample was generated using geographic area code. No geographic information was available for the mobile phone sampling frame. For the landline sample, respondent selection was the person in the household aged 18 years or older who had the “next birthday”. For the mobile sample, the person who answered was asked to participate. In 2016, the split was 60:40 between mobile and landline; the corresponding proportions were 70:30 in 2018 to take into account increases in the mobile-only population [36]. To maximize sample yield, up to six call backs were made to contact a selected number/household with calls spread over a range of days and times. Data were anonymized and no identifying information was retained in the dataset.

### Questionnaire

Details of questionnaire development have been previously described in detail elsewhere [10]. In brief, we drew questions from a previous government survey [37] and formative qualitative focus groups. The questionnaire was cognitively tested to ensure that the language was acceptable and that questions were being understood as intended with adjustments to wording made as required. Questions covered spending priorities on health; value of, barriers to, and responsibility for health prevention; attitudes to specific government-initiated interventions for health; personal health status; and demographic information. A copy of the 2016 questionnaire, with question numbers corresponding to the data items used below and in the Results may be found in the Additional File 1.

### Data items and measures

#### Demographic covariates

Age was collected as a continuous variable, or if the respondent refused, age categories were offered. For analysis, the three cut-points for age (< 35 years, 35 to < 55 years, 55+ years) reflect young, middle-age, and older-age adults, corresponding to changes in personal risk for chronic disease after 35 years [38], life stages (e.g., working, child-rearing) [39, 40] and previous research demonstrating different levels of support by age [33, 34]. Education was recoded into three categories (i.e., up to high school, post-secondary and university degree). Socioeconomic status was generated using the Index for Relative Disadvantage [41] as determined by postcode of residence and recoded into a dichotomous variable with respondents in quintiles 1 and 2 designated as disadvantaged and quintiles 3–5 as not disadvantaged.

#### Responsibility for health (E1)

Respondents were asked “To what extent do you think each of the following have a role in maintaining people’s health?” indicating their response on a 5-point Likert scale from 1 “no role at all” to 5 “a very large role”. Groups and organizations included the government, parents, and people themselves, as well as schools and food manufacturers (see Question E1, Additional file 1).

#### Specific interventions (E2)

A series of 12 statements gauged whether respondents felt the level of involvement in health for specific interventions by government was “too much”, “not enough”, or “about right”, both in general and in relation to specific policy interventions (see Question E2, Additional file 1 for specific wording of policies). Interventions and/or policies were a mix of NCD-relevant options of regulatory, fiscal, and environmental strategies which were already in place (e.g., tobacco plain packaging) [42]; could be further strengthened (e.g., alcohol advertising restrictions) [43]; have been proposed by public health advocates and experts in Australia but not yet implemented (e.g., taxing soft drink) [44]; or have been linked to health but yet to be strongly advocated (e.g., working hours) [45] as public acceptance often increases with time since implementation [33]. Compulsory immunization was included as a non-NCD prevention-related policy for comparison. As our primary interest in this analysis was to examine where there may be policy windows, responses were recoded into two categories indicating whether there was “not enough” involvement of government compared to “too much” or “about right”.

#### General attitudes towards government intervention (E3 & E5)

General attitudes to government intervention for prevention were measured by asking the question “In general, do you think Australia has too much, too little or about right amount of government regulation and policies in place to help people be healthy?” (see Question E3, Additional file 1). As with specific interventions, the response options “too much” and “about the right amount” were combined and compared with “not enough”. A series of four further questions investigated the currency of nanny state attitudes characterizing government interventions as interfering [46], paternalistic [47] or utilitarian [48] (see Question E5, Additional file 1). Response categories ranged from strongly disagree to strongly agree on a 5-point Likert scale.

### Data analysis

Design weights were calculated as the inverse of the probability of a respondent being selected to participate in the survey, accounting for the dual-frame collection methodology in which persons may have two chances of selection – one through a landline telephone and another through a mobile telephone. The weights were adjusted (calibrated) so that they matched known external benchmarks for age, gender, state and region (capital city/non-capital city), education, country of birth [49–52] and telephony status (landline only, mobile only, landline and mobile user) [53, 54] for the available year closest to the survey. Statistical analysis was undertaken using Stata version 16.1.

Demographic characteristics were tabulated and chi-square tests comparing the distributions of the 2016 and 2018 samples were performed. For each outcome measure, in order to test change from 2016 to 2018, we generated three models sequentially. Model 1 analyzed whether there was a significant change between 2016 and 2018 without covariates. Model 2 adjusted for gender, age, education and socioeconomic status, as these factors have been shown to be associated with attitudes to government regulation [33, 34] To examine whether change from 2016 to 2018 was differential across demographic characteristics, Model 3 included all two-way interactions between survey wave and each of gender, age, education and socioeconomic status. A joint test of the effect of all two-way interactions was used to examine whether the inclusion of these terms was a significant improvement on model 2.

For the regression analyses of perceptions of intervention for prevention, responses measured on a 5-point Likert scale (E1 and E5) were analyzed using linear regression to retain as much information as possible; it has been shown that parametric methods are robust for analyzing Likert scale data even where there are violations of equal variance and normal distribution assumptions [55] and in comparison to non-parametric tests [56]. Results are presented as beta coefficients representing the change in score on the 5-point scale from 2016 to 2018. Dichotomous outcomes (E2, E3) were analyzed using generalized linear models with a binomial distribution and log link with results presented as (adjusted) prevalence ratios ((A)PR).

Finally, we had observed in examining the descriptive statistics that there was not only the potential for there to be shifts in central tendency in the outcomes, but also the variation in responses (indicating increasing polarization of opinions from 2016 to 2018, perhaps not accompanied by a change in central tendency). We therefore conducted a series of one-tailed variance ratio tests which tested the hypothesis that 2018 scores showed greater variance than 2016 for all outcome variables.

## Results

A total of 2052 respondents in 2016 (response rate = 20.4%) and 2601 in 2018 (response rate = 16.7% (AAPOR RR3)) [57] were included in the sample. The (unweighted) distribution of participants’ characteristics for both surveys are shown in Table 1.

**Table 1:** Demographic profile of 2016 and 2018 AUSPOPS samples (unweighted and weighted)

Characteristic	2016		2018		2016 vs 2018
	No.	Unweighted % (weighted)	No.	Unweighted % (weighted)	*p*-value ^**c**^
Female	1092	53.2 (50.7)	1364	52.4 (50.9)	0.599
Age					0.003^**d**^
18–< 35 yrs	400	19.6 (31.4)	429	16.5 (31.5)	
35–< 55 yrs	610	29.9 (34.4)	738	28.4 (33.8)	
55 + yrs	1032	50.5 (34.3)	1432	55.1 (35.7)	
Country of birth English speaking^a^	1726	84.6 (79.2)	2183	84.0 (75.8)	0.589
English spoken at home	1750	85.6 (81.0)	2266	87.1 (80.2)	0.137
Aboriginal or Torres Strait Islander	40	2.0 (2.3)	54	2.1 (2.4)	0.770
Employment status					< 0.001^**d**^
Employed	1101	54.0 (58.6)	1343	51.8 (59.6)	
Unemployed	73	3.6 (4.2)	72	2.8 (3.8)	
Retired/pension	634	31.1 (21.7)	957	36.9 (22.9)	
Student/home duties/other	232	11.4 (15.5)	222	8.6 (13.7)	
Highest level of education					0.342
High School	648	32.4 (37.2)	832	32.8 (34.9)	
Post-secondary	616	30.8 (41.1)	822	32.4 (38.9)	
University Degree	735	36.8 (21.7)	883	34.8 (26.2)	
Disadvantaged^b^	593	29.2 (29.9)	904	35.2 (30.4)	< 0.001
Income support	666	32.8 (31.1)	864	33.4 (27.1)	0.671
Private health insurance	1305	64.2 (57.9)	1578	60.9 (55.8)	0.022

^**a**^Australia, New Zealand, United Kingdom (England, Scotland, Wales, Northern Ireland), USA, Canada

^**b**^SEIFA Index of Relative Disadvantage quintiles 1–2

^**c**^Tests performed on unweighted data

^**d**^*p*-value for omnibus tests of independence for multi-category characteristics

There were slightly more women than men in both samples. The majority of respondents were aged over 55 years and from English-speaking backgrounds, and just over half were currently employed full or part-time (Table 1). The two samples were similar for education, around a third were receiving income support, and a majority (> 60%) had private health insurance. The proportion of respondents from the most socioeconomically disadvantaged two quintiles was higher in 2018 compared with 2016. Similarly, the proportion of those aged older than 55 years and those who were retired or on a pension increased from 2016 to 2018.

### Responsibility for health (E1)

Table 2 shows descriptive statistics and results of the sequential models testing for change from 2016 to 2018, for each of the outcomes describing the size of the role of different actors and organizations in maintaining people’s health. The third and fourth columns report the effect of year of survey for the bivariate (unadjusted) and multiple variable (adjusted) analyses, respectively. The fifth column describes any interaction effects from the third set of models for any single significant (*p* < 0.10) two-way interaction term where the joint test over all interactions was statistically significant (*p* < 0.10).

**Table 2:** Weighted percentages, unadjusted and adjusted beta coefficients for responsibility for health outcomes

beta (95%CI)^**a**^ 2018 vs 2016					
To what extent do you think each of the following have a role in maintaining people’s health?	Mean score (range 1–5)^**a**^		Model 1 Unadjusted	Model 2 Adjusted^**b**^	Model 3 Interaction effect^**d**^
	2016	2018			
b) Government	3.40	3.70	0.31 (0.21, 0.40)	0.29 (0.20, 0.39)	NS
c) Parents	4.49	4.49	< 0.01 (−0.05, 0.07)	0.01 (− 0.05, 0.07)	NS
d) People themselves	4.62	4.63	< 0.01 (− 0.05, 0.06)	− 0.01 (− 0.06, 0.09)	NS
e) GPs, nurses, pharmacists	3.83	3.83	< 0.01 (−0.08, 0.08)	< 0.01 (− 0.08, 0.07)	NS
f) Employers	2.88	2.99	0.10 (0.02, 0.19)	0.10 (0.01, 0.18)^c^	Women’s scores increased comparative to men
g) Food manufacturers	3.71	3.80	0.09 (< 0.01, 0.18)	0.08 (−0.01, 0.17)	NS
h) Schools	3.93	3.94	< 0.01 (−0.07, 0.08)	<−0.01 (− 0.08, 0.07)^c^	Women’s scores increased comparative to men
i) Private health insurers	3.06	3.16	0.10 (0.01, 0.19)	0.10 (0.01, 0.19)^c^	35- < 55 years increased comparative to 18- < 35 years; 55+ stable. Low SES scores decreased comparative to med/high SES

^**a**^A higher score indicates larger role

^**b**^Adjusted for gender, age, education and area level disadvantage

^**c**^Adjusted beta coefficient without two-way interactions in the model

^**d**^Results for two-way interactions significant at *p* ≤ 0.10 when overall test for all two-way interactions significant at *p* ≤ 0.10

There was a significant increase between 2016 and 2018 in the size of the perceived role for government, employers, food manufacturers and private health insurers in maintaining people’s health (non-significant for food manufacturers once adjusted for demographic variables) (Table 2).

The largest effect was for the role of government, showing an adjusted increase of 0.31 units on a scale of 1 to 5 from 2016 to 2018. This corresponded to an (unadjusted) increase from 46 to 60% in the proportion of respondents indicating they felt that government had a large or very large role from 2016 to 2018 (Additional file 2). The joint test of the interactions was not significant indicating that the change between 2016 and 2018 was uniform across demographic subgroups.

In contrast, the effect of year was modified by a number of demographic variables for the role of employers, schools and private health insurers (Additional file 4). In detail, women increased their endorsement of a larger role for these actors relative to men from 2016 to 2018 (employers: beta = 0.23, *p* = 0.007; schools: beta = 0.28, *p* < 0.001; Additional file 3 and panels A and B, respectively, in Additional file 4). Those aged 35 to < 55 years increased their endorsement of a larger role for private health insurers compared to 18 to < 35 years, but low SES respondents showed relatively lower endorsement in 2018 compared with 2016 (panels C and D, respectively, in Additional file 4). As there was a significant change in the proportion of respondents holding private health insurance between 2016 and 2018, supplementary analysis including private health insurance in the model was conducted. The results showed that despite being highly significant (beta = 0.49, *p* < 0.001) there was no change in the direction and significance of the main effects and interactions found in the reported analyses.

The variance ratio analyses which tested whether responses had become more polarized over time showed a significant increase in variability from 2016 to 2018 for the roles for “people themselves” (*p* = 0.003), “GPs, nurses and pharmacists” (*p* = 0.017), and “private health insurers” (*p* = 0.026) (Additional file 2).

### Support for specific interventions (E2)

Support for further government action increased between 14 and 23% from 2016 to 2018 both in general and for the specific interventions of bans on smoking in cars with children, lower speed limits in high pedestrian areas, and laws setting limits on working hours; the largest adjusted relative change was for lower speed limits in pedestrian areas (14%, Table 3). The relative proportion saying that the government had not gone far enough also dropped for two interventions (setting salt limits on processed foods and compulsory immunisation at school entry), by 9 and 12%, respectively, with little change from the unadjusted to adjusted analyses.

**Table 3:** Weighted percentages, unadjusted and adjusted prevalence ratios (APR) for support for specific interventions

APR (95%CI)^**a**^ 2018 vs 2016					
Intervention	% “not far enough”^**a**^ 2016 2018		Model 1 Unadjusted	Model 2 Adjusted^**b**^	Model 3 Interaction effect^**d**^
a) Plain packaging for tobacco products	29.8%	31.8%	1.06 (0.95, 1.19)	1.06 (0.95, 1.19)	NS
b) Bans on smoking in cars with children	42.8%	48.6%	1.13 (1.05, 1.23)	1.14 (1.05, 1.23)	NS
c) Lower speed limits (30 km/hr) in high pedestrian areas	14.5%	17.9%	1.23 (1.05, 1.45)	1.23 (1.04, 1.45)	NS
d) Restrictions on advertising unhealthy foods to children	58.4%	58.6%	1.00 (0.94, 1.07)	1.00 (0.94, 1.06)^c^	Proportion of women increased comparative to men. 35- < 55 years decreased comparative to 18 < − 35 years. University and post-secondary education increased comparative to high school education
e) Restrictions on alcohol advertising	45.4%	42.9%	0.94 (0.87, 1.03)	0.93 (0.87, 1.01)	NS
f) Taxing soft drink	42.5%	43.9%	1.03 (0.95, 1.13)	1.02 (0.94, 1.10)^c^	Proportion of women increased comparative to men. Proportion of university educated decreased relative to high school
h) Setting salt limits on processed food	55.3%	50.5%	0.91 (0.85, 0.98)	0.91 (0.85, 0.97)	NS
j) Compulsory immunisation at school entry	36.3%	31.3%	0.86 (0.78, 0.96)	0.88 (0.80, 0.98)	NS
k) Laws setting limits on working hours	22.1%	25.6%	1.16 (1.01, 1.33)	1.16 (1.01, 1.34)	NS
l) Creation of bike lanes separated from cars	41.3%	44.1%	1.07 (0.98, 1.16)	1.07 (0.98, 1.16)	NS

^**a**^Responses to “For each of the following government initiatives, please tell me whether you think it shows the government going too far, not far enough or having about the right amount of involvement in helping people be healthy?”, showing percent/APR responding “Not far enough” vs combined “too far” and “about the right amount”

^**b**^Adjusted for gender, age, education and area level disadvantage

^**c**^Adjusted beta coefficient without two-way interactions in the model

^**d**^Results for two-way interactions significant at *p* ≤ 0.10 when overall test for all two-way interactions significant at *p* ≤ 0.10

Restrictions on advertising unhealthy foods to children showed a significant joint effect, including all two-way interactions (*p* = 0.050). The proportion who felt restrictions on advertising unhealthy foods to children had not gone far enough increased for women comparative to men (APR = 1.12, *p* = 0.066; Additional file 3 and panel A, Additional file 5) and those with a post-secondary (APR = 1.18, *p* = 0.025) or university education (APR = 1.13, *p* = 0.098) versus those with a high school education (Additional file 3 and panel B, Additional file 5). However, this proportion decreased for those aged between 35 and less than 55 years compared with those aged 18 to < 35 years (APR = 0.84, *p* = 0.067; Additional file 3 and panel C, Additional file 5).

There was also a significant joint effect for the interaction terms in support for taxing soft drink (*p* = 0.009). The proportion of women feeling like the government had not gone far enough on taxing soft drink had increased 2016 to 2018 compared with men (APR = 1.26, *p* = 0.004; Additional file 3 and panel D, Additional file 5). However, the proportion of people with a university education who felt this way decreased from 2016 to 2018 while it remained stable among those with high school education (*p* = 0.099; Additional file 3 and panel E, Additional file 5).

According to the variance ratio tests, the variation in responses for all of these statements in did not increase in 2018 compared with 2016 (Additional file 6).

### General attitudes towards government intervention (E3 & E5)

Only one question, which asked respondents’ agreement with the statement “Sometimes government needs to make laws that keep people from harming themselves”, showed significant change between 2016 and 2018 (Table 4). The effect was modified by demographic variables (joint test of interactions *p* = 0.068) with both the wave by age (*p* = 0.069) and wave by education (*p* = 0.055) interactions significant (Additional file 3). Specifically, relative to those aged 18 to < 35 years, those aged 55 years and older increased their agreement with this statement (beta = 0.22, *p* = 0.037; Additional file 3 and panel A, Additional file 7); those with a post-secondary education decreased their agreement relative to those with a high school education (beta = − 0.24, *p* = 0.024; Additional file 3 and panel B, Additional file 7). The proportion of people feeling Australia in general does not have enough government intervention (relative to too much or about the right amount combined) showed a significant (adjusted) relative increase of 16% between 2016 and 2018 (Table 3).

**Table 4:** Weighted percentages, unadjusted and adjusted beta coefficients for perceptions of government intervention for health

beta (95%CI)^**a**^ 2018 vs 2016					
**Do you agree or disagree with the following statements?**	**Mean score (range 1–5)**^**a**^		**Model 1** **Unadjusted**	**Model 2** **Adjusted**^**b**^	**Model 3** **Interaction effect**^**d**^
	**2016**	**2018**			
**a) Sometimes government needs to make laws that keep people from harming themselves**	3.83	3.93	0.10 (0.02, 0.19)	0.10 (0.01, 0.18)^c^	55+ years increased agreement 2016 to 2018 relative to 18–35 years Post-secondary education decreased agreement compared with high school educated
**b) The government interferes far too much in our everyday lives**	3.04	2.99	−0.05 (−0.14, 0.05)	−0.02 (−0.12, 0.08)	NS
**c) It’s not the government’s business to try to protect people from themselves**	3.04	2.96	−0.09 (− 0.18, 0.01)	−0.06 (− 0.16, 0.03)	NS
**d) Government should put limits on the choices individuals can make so they don’t get in the way of what’s good for society**	2.69	2.70	0.01 (−0.09, 0.11)	0.01 (− 0.09, 0.11)	NS
				**APR (95%CI)**^**a**^ **2018 vs 2016**	
	**% Not enough**^**e**^		**Model 1**	**Model 2**	**Model 3**
	**2016**	**2018**	**Unadjusted**	**Adjusted**^b^	**Interaction effect**^c^
**E3 In general, do you think Australia has too much, too little or about the right amount of government regulation and policies in place to help people be healthy?**	43.9%	50.4%	1.15 (1.06, 1.25)	1.16 (1.07, 1.26)	NS

^a^A higher score indicates greater agreement with the statement

^b^Adjusted for gender, age, education and area level disadvantage

^c^Adjusted beta coefficient without two-way interactions in the model

^d^Results for two-way interactions significant at *p* ≤ 0.10 when overall test for all two-way interactions significant at *p* ≤ 0.10

^e^Not far enough vs “too far” and “about the right amount” combined

In contrast to the above results, the variance ratio tests were highly significant for all four of the perceptions of government intervention (*p* < 0.001 for all questions) indicating greater polarization of opinions across these statements in 2018 compared with 2016 (Additional file 8).

## Discussion

Despite the short intervening period, our two surveys of community perceptions showed significant change both in terms of level of support for government intervention, as well as the degree of polarization of opinions. We observed differences in support for intervention in general and for specific interventions, including for interventions already in place (e.g., bans on smoking in cars with children) and those yet to be implemented (e.g., sugar tax). Our analyses also showed that change was not always uniform across demographic subgroups, with the degree or direction of change varying by gender, age and education for a number of measures. The analyses point to a multilayered picture of increasing appetite for government leadership on prevention and greater disparity between subpopulations in opinion. These findings are discussed in greater detail below in the context of previous research along with implications for public health policy practice and research.

One of the strongest effects was the almost 14% absolute change from 46% in 2016 to 60% in 2018 in the proportion of people saying that government has a large or very large role in maintaining people’s health. While endorsement of a larger role for employers and private health insurers also increased significantly, the change was considerably smaller. In keeping with this result, support for three specific interventions also increased from 2016 to 2018, as did agreement that the government needs laws to stop people from harming themselves and that, in general, there was not enough regulation and policies in place to help people be healthy. Thus, there appears to be a gap between the role that the community perceives the government should be taking in prevention, and the perception that that role is being carried out. While the mechanism producing such changes is difficult to determine from our data, there is current evidence in the COVID-19 [58] other health literature [59] of strong public acceptance of government intervention among Australians. It also appears that the increasing and majority support for government to lead on prevention seen in our study is apparently compatible with strong (but stable) endorsement of personal responsibility for health, which was unchanged between 2016 and 2018. Concomitantly, there is little evidence of community concerns of a “nanny state”, confirming a previous analysis of the 2016 AUSPOPS data [10].

Looking across support for specific interventions, no clear pattern emerged in the types of interventions where change was observed. There was, however, continuing strong support for greater restrictions on advertising unhealthy foods to children; support remained favorable (58%) and stable from 2016 to 2018 despite some subpopulations (i.e., women, people aged younger than 35, and those with a university education) increasingly feeling that the government had not gone far enough. In Australia, restrictions on unhealthy food advertising are currently under self-regulation [60, 61], a policy which has been criticized by health advocates and researchers for being ineffective in reducing children’s exposure over the past decade [62, 63]. As government regulation is yet to be implemented in Australia and is enjoying a surge of support in some groups, a policy change is likely to be met with public endorsement, thereby satisfying the political stream of Kingdon’s policy window [16]. However, as with many other preventive health interventions [33, 34], women are more (and increasingly) supportive of this intervention compared with men, and therefore, any accompanying advocacy would need to address the differential endorsement within subpopulations and address the concerns of groups whose support is on the wane. One solution may be to communicate the effectiveness of such policy interventions which has been shown to increase support in the past [64]. Importantly, it is not clear whether this strategy appeals to some subpopulations over others and would be a key area to explore in future research.

The decrease between the two surveys in the proportion feeling that the government had not gone far enough for a couple of the interventions may reflect changes in the regulatory environment in this period. For example, the reduction between 2016 and 2018 in the proportion saying that compulsory immunization at school entry had not gone far enough could be a result of the introduction of amendments to the “No Jab No Pay” policy in 2016. The legislation principally restricts eligibility to some family and child care tax benefits if children are not immunized, and in 2016 the exemption for conscientious objectors was removed [65]. Survey research conducted post-implementation demonstrated the amended legislation drew high levels of support (82%) among parents of children age younger than 5 years and immunization coverage was only 1% below the target of 95% [65]. Our findings may therefore indicate that the community feels current action is sufficient for achieving this particular health objective.

Our survey findings of significant support for regulation on salt in processed foods was consistent with other Australian research [66]. However, although the majority still felt in 2018 that the government had not gone far enough in setting limits on salt in processed foods, there had been a significant and uniform (across demographic subgroups) drop in support for change. Reformulation of the food supply is seen as a best buy for non-communicable disease prevention [67]. However, while the UK has seen success from implementation of a national salt reduction strategy which includes voluntary reformulation [68], Australia’s voluntary thresholds and actions [69] have had minimal impact [70] due to a lack of strong government leadership, targets and timelines, as well as lack of accountability for industry inaction [71]. The change in public support in our sample may signal a need for public health advocates to maintain the visibility of issues yet to be translated into policy, such as salt reduction strategies, to ensure the potential for action is not lost through a decline in public support.

Finally, a novel aspect of our analysis was to examine not only shifts in central tendency but also variation over time. While we saw only one significant change in the mean score with the different conceptualizations of government intervention in general, all scores exhibited greater polarization in opinion. Thus, while community support for specific interventions continues to trend upwards for some and downwards for others, positions on the spectrum of different conceptualizations of how government should act out its role in general is balanced but becoming more strongly held in both directions. Our results confirm the capacity for dissonance between positions on intervention in general and for specific interventions, as hypothesized in previous AUSPOPS research [10]. More broadly, research on the question of whether social opinion is becoming more polarized due to the reinforcing nature of personalized news and information, especially through social media, has shown mixed results [72–74]. While we cannot test this assumption with our data, future research may benefit from gauging the extent of respondents’ use of social media and other information sources to investigate potential relationship between engagement with more personalized news streams and strength of opinion. Such analyses could also further inform policymakers how best to reach different subpopulations with health information according to issue and the attitude held.

### Strengths and limitations

This study has a number of strengths. First, the samples from both years are nationally representative, were sampled in a comparable manner and are of sufficient size to allow for subgroup analyses. Second, the survey includes questions which address both positions on intervention generally as well as for specific interventions and used the same wording for the two comparison years. Limitations include some small demographic differences between the two samples on age, employment and holding private insurance, and a lack of information on non-responders meant we could not compare those who did and did not respond to the survey. However, the analyses were weighted to the Australian population and therefore are reflective of the general population. Response rates were low but typical of telephone surveys [75, 76] and accounted for the changes in phone ownership towards greater mobile phone coverage [36]. It was also possible that respondents did not confine their interpretation of “health” (in questions E1, E3 and E5) to NCD prevention which may introduce a level of error into our conclusions. However, as stated in the Methods, questions were cognitively tested prior to administration and found to be reliable in terms of intended meaning and therefore are likely to reflect community attitudes on the prevention of lifestyle-related chronic disease. Finally, the changes noted here may not be indicative of longer-lasting shifts in opinions and may be influenced dynamically by current events. However, there was no evidence of any relevant and major controversial issues present during the survey periods.

## Conclusion and future directions

Our study demonstrates that measuring community attitudes about prevention at different time points can allow identification of change in support for specific interventions and in appetite for government intervention more generally. Moreover, the differential change in perceptions across demographic subpopulations points to where information and advocacy may need to be targeted at particular subpopulations to garner public endorsement. Such information is important for policymakers and public health advocates such that they can take advantage of support for further preventive action and contribute to the opening of policy windows that support such action. Future research should continue to monitor the permanence and/or direction of the changes identified here. Perhaps additional investigations into associations between change and exposure to information sources as well as health indicators (e.g., experience of chronic disease, weight status) if that was informative for the targeting of information and advocacy for interventions for prevention. In terms of research translation, surveys such as AUSPOPS produced through the collaborative endeavors like the Australian Prevention Partnership Centre (https://preventioncentre.org.au/) will facilitate the dissemination and use of evidence for policy change [77] advocated by current [7, 78] NCD-relevant consensus statements and plans and those in development. Such models should continue to be supported to generate policy-relevant and timely evidence to build community support for policy change.

## Supplementary Information

**Additional file 1.** AUSPOPS survey questionnaire. Full survey questionnaire 2018 AUSPOPS.

**Additional file 2. **Distribution of responses and variance ratio tests for responsibility for health (D1). Percentages and variance ratio test *p*-values for responsibility for health (D1).

**Additional file 3. **Results for models with significant joint tests of all two-way interactions supporting column 6, Tables 2, 3 and 4. APRs and *p*-values for all models with significant joint tests of two-way interactions.

**Additional file 4.** Predicted adjusted margins for the significant interactions between wave and demographic variables for responsibility for health outcomes. Figures showing adjusted predicted adjusted margins for significant two-way interactions for models with significant joint tests of two-way interactions for responsibility for health outcomes (D1).

**Additional file 5.** Predicted adjusted margins for the significant interaction between wave and demographic variables for specific interventions. Figures showing adjusted predicted adjusted margins for significant two-way interactions for models with significant joint tests of two-way interactions for specific interventions (E2).

**Additional file 6. **Distribution of responses for support for specific interventions (E2). Percentages and variance ratio test *p*-values for specific interventions (E2).

**Additional file 7.** Predicted adjusted margins for the significant interactions between wave and demographic variables for general attitudes towards government intervention. Figures showing adjusted predicted adjusted margins for significant two-way interactions for models with significant joint tests of two-way interactions for general attitudes towards government intervention (E3 & E5).

**Additional file 8. **Distribution of responses for perceptions of government intervention for health (E5, E3). Percentages and variance ratio test *p*-values for general attitudes towards government intervention (E3 & E5).

## Data Availability

The datasets generated and/or analysed during the current study are not publicly available due to such access would be a breach of the original ethics approval for the studies but are available from the corresponding author on reasonable request.

## Abbreviations

AAPOR: American Association for Public Opinion Research
APR: Adjusted prevalence ratio
AUSPOPS: Australian Perceptions of Prevention Survey
NS: Not significant
