# Supplementary material for: Changes in Australian community perceptions of non-communicable disease prevention: a greater role for government?
Source: BMC Public Health. 2021 Nov 15;21:2094. doi: 10.1186/s12889-021-12159-9 (PMC8591602; doi:10.1186/s12889-021-12159-9)
Supplement: Supplementary file 2 — Additional file 2. Distribution of responses and variance ratio tests for responsibility for health (D1). Percentages and variance ratio test p-values for responsibility for health (D1). [file 12889_2021_12159_MOESM2_ESM.docx]

Additional file 2: Distribution of responses and variance ratio tests for responsibility for health (D1)

| **To what extent do you think each of the following have a role in maintaining people’s health?** | **2016** | **2018** | **Variance Ratio test p-value^1^** |
| --- | --- | --- | --- |
| **Government** |  |  | 0.255 |
| No role at all | 8.1% | 7.8% |  |
| A small role | 15.0% | 9.4% |  |
| A moderate role | 30.8% | 22.1% |  |
| A large role | 21.3% | 25.9% |  |
| A very large role | 24.8% | 34.7% |  |
| **Parents** |  |  | 0.359 |
| No role at all | 1.1% | 1.4% |  |
| A small role | 1.0% | 1.9% |  |
| A moderate role | 8.7% | 7.3% |  |
| A large role | 26.2% | 24.8% |  |
| A very large role | 62.9% | 64.6% |  |
| **People themselves** |  |  | **0.003** |
| No role at all | 0.9% | 1.4% |  |
| A small role | 0.9% | 1.2% |  |
| A moderate role | 8.1% | 6.8% |  |
| A large role | 15.3% | 14.5% |  |
| A very large role | 74.9% | 76.1% |  |
| **GPs, nurses, pharmacists** |  |  | **0.017** |
| No role at all | 2.0% | 2.6% |  |
| A small role | 7.0% | 7.1% |  |
| A moderate role | 27.7% | 27.7% |  |
| A large role | 32.6% | 29.9% |  |
| A very large role | 30.7% | 32.7% |  |
| **Employers** |  |  | 0.593 |
| No role at all | 12.6% | 10.8% |  |
| A small role | 23.7% | 19.7% |  |
| A moderate role | 36.1% | 40.6% |  |
| A large role | 18.1% | 18.0% |  |
| A very large role | 9.5% | 11.0% |  |
| **Food manufacturers** |  |  | 0.955 |
| No role at all | 6.2% | 4.8% |  |
| A small role | 10.7% | 9.7% |  |
| A moderate role | 21.5% | 21.8% |  |
| A large role | 28.6% | 27.9% |  |
| A very large role | 33.0% | 35.9% |  |
| **Schools** |  |  | 0.202 |
| No role at all | 2.3% | 2.9% |  |
| A small role | 4.8% | 6.3% |  |
| A moderate role | 24.0% | 20.9% |  |
| A large role | 34.8% | 34.0% |  |
| A very large role | 34.1% | 36.0% |  |
| **Private health insurers** |  |  | **0.026** |
| No role at all | 12.4% | 12.5% |  |
| A small role | 19.6% | 17.2% |  |
| A moderate role | 34.0% | 31.1% |  |
| A large role | 17.6% | 20.2% |  |
| A very large role | 16.4% | 18.9% |  |

One tailed test of variance ratio 2016/2018 <1.0
