# Supplementary material for: Changes in Australian community perceptions of non-communicable disease prevention: a greater role for government?
Source: BMC Public Health. 2021 Nov 15;21:2094. doi: 10.1186/s12889-021-12159-9 (PMC8591602; doi:10.1186/s12889-021-12159-9)
Supplement: Supplementary file 3 — Additional file 3. Results for models with significant joint tests of all two-way interactions supporting column 6, Tables 2, 3 and 4. APRs and p-values for all models with significant joint tests of two-way interactions. [file 12889_2021_12159_MOESM3_ESM.docx]

Additional file 3: Results for models with significant joint tests of all two-way interactions supporting column 6, Tables 2-4

|  | **Responsibility for health (Table 2)**  **beta (95%CI)**  **(raw score range 1-5)** | | | **Support for specific interventions (Table 3)**  **APR (95%CI)**  **(“Not far enough” vs “too far”/“about the right amount**”) | | **Perceptions of government intervention**  **(Table 4)**  **beta (95%CI)**  **(raw score range 1-5)** |
| --- | --- | --- | --- | --- | --- | --- |
| **Effect (reference category)** | **Employers** ^c^ | **Schools** ^c^ | **Private health insurers** ^c^ | **Unhealthy ads for children** ^c^ | **Soft drink tax** ^c^ | **Make laws that keep people from harming themselves** ^c^ |
| **All 2-way interactions** | **p=0.091** | **p=0.025** | **p=0.073** | **p=0.050** | **p=0.009** | **p=0.068** |
| **Wave** (2016) |  |  |  |  |  |  |
| 2018 | -0.03 (-0.26, 0.21) | -0.11 (-0.33, 0.10) | 0.12 (-0.12, 0.37) | 0.93 (0.76, 1.13) | 0.92 (0.70, 1.21) | 0.15 (-0.08, 0.37) |
| **Gender** (male) |  |  |  |  |  |  |
| Female | 0.09 (-0.04, 0.22) | -0.09 (-0.2, 0.02) | 0.13 (0.00, 0.27) | 0.95 (0.87, 1.03) | 0.97 (0.87, 1.09) | 0.15 (0.04, 0.26) |
| Wave by gender | **0.23 (0.06, 0.40)** | **0.28 (0.13, 0.43)** | 0.11 (-0.08, 0.30) | **1.12 (0.99, 1.25)** | **1.26 (1.08, 1.47)** | -0.04 (-0.21, 0.12) |
| **Age** (<35 years) |  |  |  |  |  |  |
| 35-<55yrs | 0.02 (-0.15, 0.19) | -0.14 (-0.29, 0.00) | -0.20 (-0.38, -0.02) | 1.39 (1.21, 1.60) | 1.43 (1.18, 1.72) | -0.08 (-0.22, 0.06) |
| 55+yrs | 0.13 (-0.02, 0.29) | -0.13 (-0.27, 0.00) | 0.25 (0.09, 0.42) | 1.42 (1.24, 1.62) | 1.57 (1.32, 1.87) | -0.26 (-0.40, -0.12) |
| Wave by age | p=0.90 | p=0.867 | p=0.040 | p=0.098 | p=0.743 | p=0.069 |
| Wave by 35-<55yrs | 0.04 (-0.19, 0.27) | 0.01 (-0.20, 0.22) | **0.22 (-0.03, 0.48)** | **0.84 (0.69, 1.01)** | 0.97 (0.75, 1.26) | 0.04 (-0.18, 0.26) |
| Wave by 55+yrs | 0.07 (-0.14, 0.29) | 0.04 (-0.14, 0.23) | -0.05 (-0.28, 0.18) | 0.94 (0.79, 1.12) | 1.04 (0.81, 1.32) | **0.22 (0.01, 0.42)** |
| **Education** |  |  |  |  |  |  |
| Post-secondary | -0.04 (-0.19, 0.12) | 0.05 (-0.08, 0.18) | -0.11 (-0.27, 0.05) | 0.92 (0.82, 1.02) | 1.01 (0.86, 1.18) | -0.02 (-0.15, 0.12) |
| Uni Degree | -0.15 (-0.30, -0.01) | -0.07 (-0.19, 0.05) | -0.14 (-0.3, 0.01) | 1.01 (0.92, 1.12) | 1.36 (1.19, 1.56) | 0.12 (-0.01, 0.25) |
| Wave by education | p=0.372 | p=0.843 | p=0.285 | p=0.070 | p=0.099 | p=0.055 |
| Wave by post-secondary | -0.08 (-0.29, 0.13) | -0.06 (-0.25, 0.14) | -0.1 (-0.32, 0.13) | **1.18 (1.02, 1.37)** | 1.01 (0.82, 1.24) | **-0.24 (-0.45, -0.03)** |
| Wave by uni degree | 0.06 (-0.14, 0.26) | -0.01 (-0.19, 0.16) | -0.18 (-0.4, 0.04) | **1.13 (0.98, 1.30)** | **0.84 (0.70, 1.02)** | -0.05 (-0.25, 0.14) |
| **SES** (high SES^a^) |  |  |  |  |  |  |
| Disadvantaged^b^ | 0.03 (-0.10, 0.17) | -0.02 (-0.14, 0.10) | 0.01 (-0.15, 0.16) | 0.92 (0.83, 1.01) | 0.81 (0.70, 0.93) | -0.03 (-0.16, 0.09) |
| Wave by disadvantaged^b^ | -0.06 (-0.25, 0.12) | -0.07 (-0.24, 0.10) | **-0.2 (-0.41, 0.02)** | 1.04 (0.91, 1.19) | 1.11 (0.91, 1.35) | -0.03 (-0.22, 0.16) |

^a^ SEIFA Index of relative advantage-disadvantage quintiles 3-5

^b^ SEIFA Index of relative advantage-disadvantage quintiles 1-2

^c^ Bolded result, two-way interaction significant at p<0.10
