# Supplementary material for: Changes in Australian community perceptions of non-communicable disease prevention: a greater role for government?
Source: BMC Public Health. 2021 Nov 15;21:2094. doi: 10.1186/s12889-021-12159-9 (PMC8591602; doi:10.1186/s12889-021-12159-9)
Supplement: Supplementary file 4 — Additional file 4. Predicted adjusted margins for the significant interactions between wave and demographic variables for responsibility for health outcomes. Figures showing adjusted predicted adjusted margins for significant two-way interactions for models with significant joint tests of two-way interactions for responsibility for health outcomes (D1). [file 12889_2021_12159_MOESM4_ESM.docx]

Additional file 4: Predicted adjusted margins for the significant interactions between wave and demographic variables for responsibility for health outcomes

B

A

C

D

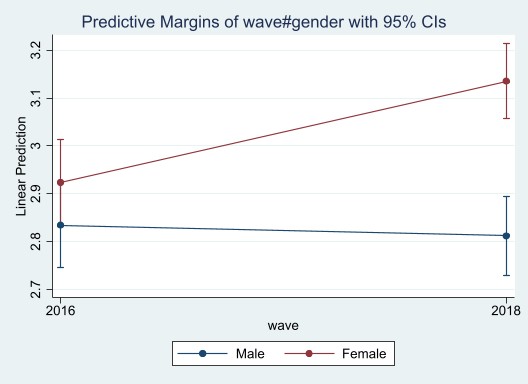

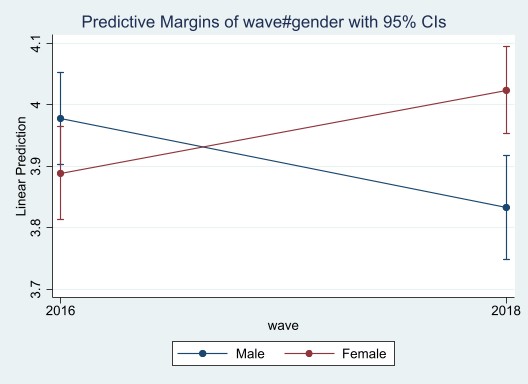

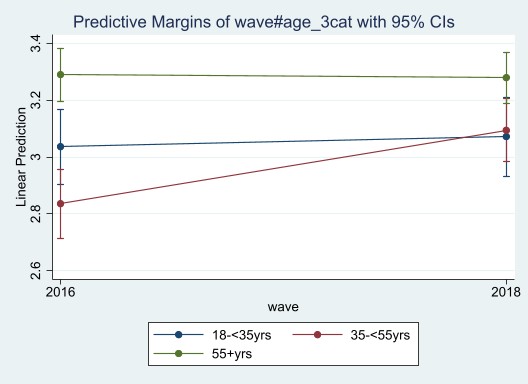

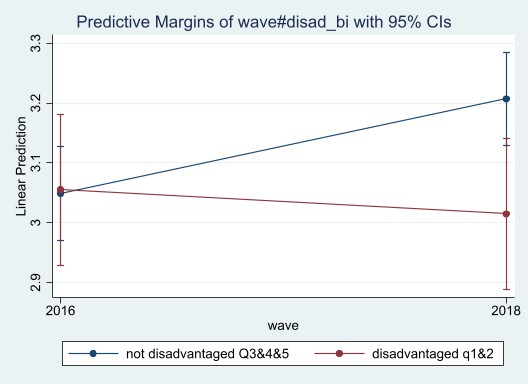


Panel A shows predicted adjusted margins for the interaction between wave and gender for the size of the role of employers

Panel B shows predicted adjusted margins for the interaction between wave and gender for the size of the role of schools

Panel C shows predicted adjusted margins for the interaction between wave and age for the size of the role of private health insurers

Panel D shows predicted adjusted margins for the interaction between wave and socioeconomic disadvantage for the size of the role of private health insurers
