# Supplementary material for: Changes in Australian community perceptions of non-communicable disease prevention: a greater role for government?
Source: BMC Public Health. 2021 Nov 15;21:2094. doi: 10.1186/s12889-021-12159-9 (PMC8591602; doi:10.1186/s12889-021-12159-9)
Supplement: Supplementary file 5 — Additional file 5. Predicted adjusted margins for the significant interaction between wave and demographic variables for specific interventions. Figures showing adjusted predicted adjusted margins for significant two-way interactions for models with significant joint tests of two-way interactions for specific interventions (E2). [file 12889_2021_12159_MOESM5_ESM.docx]

Addition file 5: Predicted adjusted margins for the significant interaction between wave and demographic variables for specific interventions


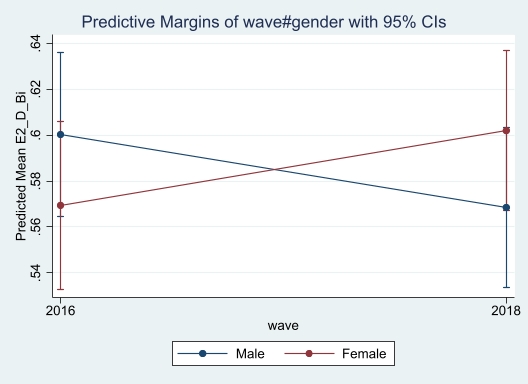

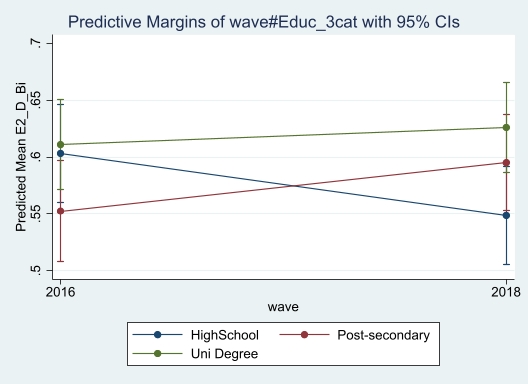

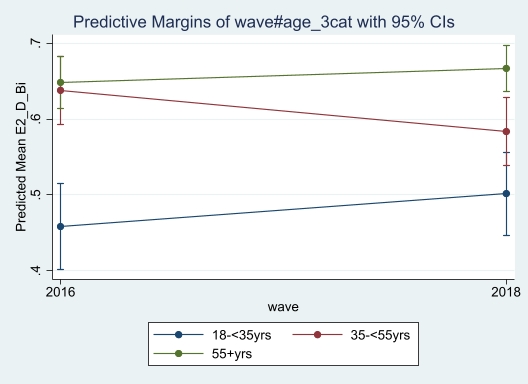

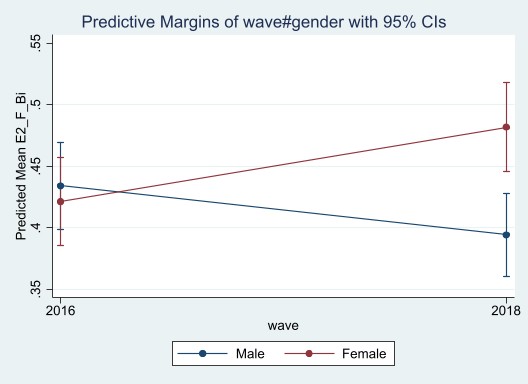

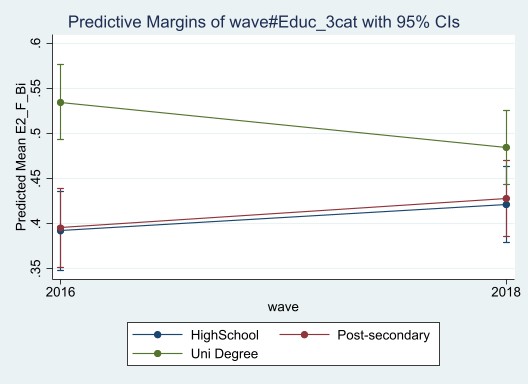


B

A

C

D

E

Panel A shows predicted adjusted margins for the interaction between wave and gender of proportion saying the government had not gone far enough on restricting advertising of unhealthy foods to children.

Panel B shows predicted adjusted margins for the interaction between wave and education of proportion saying the government had not gone far enough on restricting advertising of unhealthy foods to children.

Panel C shows predicted adjusted margins for the interaction between wave and age of proportion saying the government had not gone far enough on restricting advertising of unhealthy foods to children

Panel D shows predicted adjusted margins for the interaction between wave and gender of proportion saying the government had not gone far enough on taxing soft drink.

Panel E shows predicted adjusted margins for the interaction between wave and education of proportion saying the government had not gone far enough on taxing soft drink.
