# Supplementary material for: Changes in Australian community perceptions of non-communicable disease prevention: a greater role for government?
Source: BMC Public Health. 2021 Nov 15;21:2094. doi: 10.1186/s12889-021-12159-9 (PMC8591602; doi:10.1186/s12889-021-12159-9)
Supplement: Supplementary file 6 — Additional file 6. Distribution of responses for support for specific interventions (E2). Percentages and variance ratio test p-values for specific interventions (E2). [file 12889_2021_12159_MOESM6_ESM.docx]

#### Additional file 6: Distribution of responses for support for specific interventions (E2)

| **Intervention** | **2016** | **2018** | **Variance Ratio test p-value^1^** |
| --- | --- | --- | --- |
| **Plain packaging for tobacco products** |  |  | 0.627 |
| Too far | 15.4% | 12.7% |  |
| About the right amount | 54.8% | 55.5% |  |
| Not far enough | 29.8% | 31.8% |  |
| **Bans smoking cars with children** |  |  | 0.537 |
| Too far | 4.8% | 4.0% |  |
| About the right amount | 52.4% | 47.4% |  |
| Not far enough | 42.8% | 48.6% |  |
| Total | 100.0% | 100.0% |  |
| **Lower speed limits (30km/hr) in high pedestrian areas** |  |  | 0.944 |
| Too far | 26.8% | 15.9% |  |
| About the right amount | 58.6% | 66.2% |  |
| Not far enough | 14.5% | 17.9% |  |
| **Restrictions on advertising unhealthy foods to children** | |  | 0.975 |
| Too far | 6.5% | 5.5% |  |
| About the right amount | 35.1% | 36.0% |  |
| Not far enough | 58.4% | 58.6% |  |
| **Restrictions on alcohol advertising** |  |  | 0.741 |
| Too far | 7.6% | 8.1% |  |
| About the right amount | 47.0% | 49.0% |  |
| Not far enough | 45.4% | 42.9% |  |
| **Taxing soft drink** |  |  | 0.851 |
| Too far | 22.9% | 20.1% |  |
| About the right amount | 34.6% | 36.0% |  |
| Not far enough | 42.5% | 43.9% |  |
| **Setting salt limits on processed food** |  |  | 0.351 |
| Too far | 8.5% | 7.5% |  |
| About the right amount | 36.2% | 42.1% |  |
| Not far enough | 55.3% | 50.5% |  |
| **Compulsory immunization at school entry** |  |  | 0.878 |
| Too far | 8.2% | 7.5% |  |
| About the right amount | 55.5% | 61.2% |  |
| Not far enough | 36.3% | 31.3% |  |
| **Laws setting limits on working hours** |  |  | 0.753 |
| Too far | 16.4% | 14.8% |  |
| About the right amount | 61.5% | 59.6% |  |
| Not far enough | 22.1% | 25.6% |  |
| **Creation of bike lanes separated from cars** |  |  | 0.233 |
| Too far | 10.4% | 9.6% |  |
| About the right amount | 48.3% | 46.3% |  |
| Not far enough | 41.3% | 44.1% |  |

^1^ One tailed test of variance ratio 2016/2018 <1.0
