# Supplementary material for: Changes in Australian community perceptions of non-communicable disease prevention: a greater role for government?
Source: BMC Public Health. 2021 Nov 15;21:2094. doi: 10.1186/s12889-021-12159-9 (PMC8591602; doi:10.1186/s12889-021-12159-9)
Supplement: Supplementary file 7 — Additional file 7. Predicted adjusted margins for the significant interactions between wave and demographic variables for general attitudes towards government intervention. Figures showing adjusted predicted adjusted margins for significant two-way interactions for models with significant joint tests of two-way interactions for general attitudes towards government intervention (E3 & E5). [file 12889_2021_12159_MOESM7_ESM.docx]

Additional file 7: Predicted adjusted margins for the significant interactions between wave and demographic variables for general attitudes towards government intervention

B

A


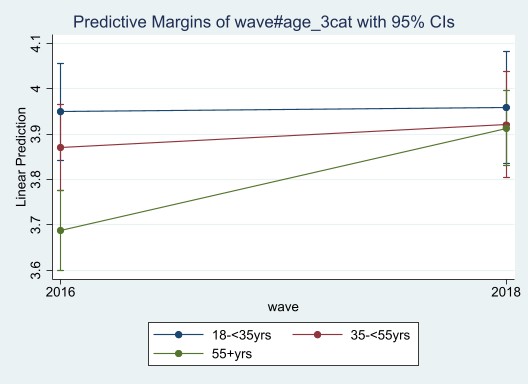

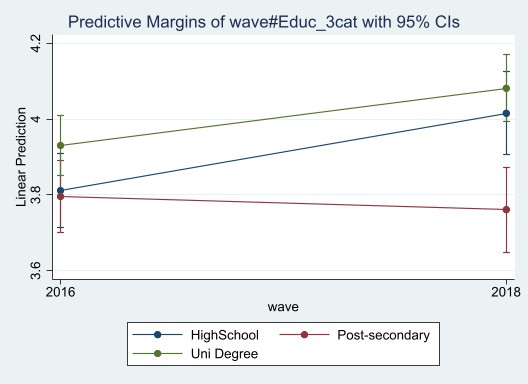


Panel A shows predicted adjusted margins for the interaction between wave and age of agreement with the statement that “sometimes government needs to make laws that keep people from harming themselves”.

Panel B shows predicted adjusted margins for the interaction between wave and education of agreement with the statement that “sometimes government needs to make laws that keep people from harming themselves”.
