# Supplementary material for: Changes in Australian community perceptions of non-communicable disease prevention: a greater role for government?
Source: BMC Public Health. 2021 Nov 15;21:2094. doi: 10.1186/s12889-021-12159-9 (PMC8591602; doi:10.1186/s12889-021-12159-9)
Supplement: Supplementary file 8 — Additional file 8. Distribution of responses for perceptions of government intervention for health (E5, E3). Percentages and variance ratio test p-values for general attitudes towards government intervention (E3 & E5). [file 12889_2021_12159_MOESM8_ESM.docx]

Additional file 8: Distribution of responses for perceptions of government intervention for health (E5, E3)

| **Statement** | **2016** | **2018** | **Variance Ratio test p-value^1^** |
| --- | --- | --- | --- |
| **a) Sometimes government needs to make laws that keep people from harming themselves** |  |  | **<0.001** |
| Strongly disagree | 4.4% | 6.3% |  |
| Disagree | 12.8% | 11.4% |  |
| Neither agree nor disagree | 3.2% | 1.2% |  |
| Agree | 55.0% | 44.9% |  |
| Strongly agree | 24.7% | 36.1% |  |
| **b) The government interferes far too much in our everyday lives** |  |  | **<0.001** |
| Strongly disagree | 6.0% | 10.2% |  |
| Disagree | 42.6% | 42.8% |  |
| Neither agree nor disagree | 8.1% | 4.1% |  |
| Agree | 28.4% | 23.3% |  |
| Strongly agree | 14.9% | 19.5% |  |
| **c) It’s not the government's business to try to protect people from themselves** |  |  | **<0.001** |
| Strongly disagree | 9.8% | 15.1% |  |
| Disagree | 37.4% | 36.8% |  |
| Neither agree nor disagree | 5.0% | 3.3% |  |
| Agree | 34.3% | 27.2% |  |
| Strongly agree | 13.5% | 17.7% |  |
| **d) Government should put limits on the choices individuals can make so they don't get in the way of what's good for society** |  |  | **<0.001** |
| Strongly disagree | 18.2% | 23.0% |  |
| Disagree | 38.9% | 33.5% |  |
| Neither agree nor disagree | 5.9% | 3.5% |  |
| Agree | 29.9% | 30.6% |  |
| Strongly agree | 7.1% | 9.4% |  |
| **E3 In general, do you think Australia has too much, too little or about the right amount of government regulation and policies in place to help people be healthy?** |  |  | 0.122 |
| Too much | 9.2% | 9.2% |  |
| About the right amount | 47.0% | 40.4% |  |
| Not enough | 43.9% | 50.4% |  |

^1^ One tailed test of variance ratio 2016/2018 <1.0
